# Friendship and momentary loneliness in dementia caregiving: daily experiences of caregivers with high and low burden

**DOI:** 10.1093/geronb/gbaf190

**Published:** 2025-10-01

**Authors:** Yee To Ng, Angela Turkelson, Anna Kratz, Kira Birditt

**Affiliations:** Institute for Social Research, University of Michigan, Ann Arbor, Michigan, United States; Institute for Social Research, University of Michigan, Ann Arbor, Michigan, United States; Department of Physical Medicine and Rehabilitation, University of Michigan, Ann Arbor, Michigan, United States; Institute for Social Research, University of Michigan, Ann Arbor, Michigan, United States

**Keywords:** Daily social experiences, Non-kin ties, Momentary loneliness, Caregiving stress

## Abstract

**Objectives:**

Dementia caregivers are particularly vulnerable to loneliness. Grounded in the social convoy and stress process models, this study examined whether (a) dementia caregivers with fewer close friends experience higher levels of momentary loneliness; (b) daily friend interactions are linked to reduced momentary loneliness, and whether the quality and closeness of these interactions matter; and (c) whether the link between friend interaction and momentary loneliness varies between caregivers with high versus low caregiver burden.

**Methods:**

The sample included 223 dementia caregivers (Mean_age_ = 61.38, 78% female, 36% Black) from the Stress and Well-Being in the Everyday Lives of Caregivers Study. They completed a baseline interview assessing their demographics, social network, and caregiving characteristics, followed by a 5-day ecological momentary assessment (EMA), reporting their social interactions and loneliness every 3 hr.

**Results:**

A greater number of close friends was associated with lower levels of momentary loneliness. Caregivers reported interactions with friends in 22% of EMA surveys. Multilevel linear models showed that caregivers experienced lower momentary loneliness when they interacted with friends, especially during positive interactions and with non-close friends, compared to when they did not. Furthermore, friend interactions were associated with lower momentary loneliness for caregivers with higher burden, but not for those with lower burden.

**Discussion:**

These results highlight the need for interventions that foster friendships and encourage caregivers to schedule social interactions with friends to alleviate loneliness and enhance their well-being.

Loneliness is increasingly recognized as a public health concern due to its significant impact on mental and physical health, as well as its association with higher morbidity and mortality rates (Bruss et al., 2024; Hawkley & Cacioppo, 2010; Holt-Lunstad, 2015, 2024). Caregivers are particularly vulnerable to loneliness compared to non-caregivers (Hajek et al., 2021; Peavy et al., 2022), with research showing that dementia caregivers face an even greater risk than non-dementia caregivers (Chen et al., 2025). Although all caregivers may experience reduced personal time and social interactions resulting from caregiving burden and stress, dementia caregivers face additional challenges that may *uniquely* increase their risk of loneliness. For example, the progressive cognitive decline and unpredictable behavioral changes in a care recipient (CR) with dementia require constant supervision, which can limit caregivers’ ability to leave the house, plan or attend social activities, and maintain social connections. Furthermore, as the CR changes, such as no longer recognizing the caregiver or behaving differently, the emotional connection between caregivers and the CR may diminish, leading to a unique form of loneliness tied to relational loss and feelings of powerlessness (Vasileiou et al., 2017; Wood & Suizzo, 2025). A meta-analysis of studies across various countries found that approximately half of dementia caregivers experience loneliness, with prevalence estimates ranging from 42% to 60% (Liao et al., 2024). This high prevalence is concerning, as loneliness among dementia caregivers not only affects their well-being but may also compromise the quality of care they provide. Moreover, previous research has primarily relied on cross-sectional data to assess loneliness among dementia caregivers (Liao et al., 2024), few studies have explored real-time fluctuations in “acute” loneliness within this high-risk population, highlighting a critical gap in the literature. Given that loneliness is increasingly recognized as a dynamic rather than static experience (Compernolle et al., 2021; Goldman & Compernolle, 2023; Zhaoyang et al., 2022), capturing these momentary dynamics is crucial for identifying specific contexts that may alleviate or exacerbate loneliness in caregivers’ daily lives, which, in turn, can inform the development of timely and context-sensitive interventions, tailored to caregivers’ immediate needs and vulnerable moments.

Social networks and support are essential in helping dementia caregivers through the challenges of their role. Two theoretical frameworks guide this study: the social convoy model conceptualizes individuals as moving through life surrounded by a dynamic network of social relationships that vary in closeness and provide different levels of contact and support (Antonucci et al., 2014). This model emphasizes the importance of close ties, including friendships, that can serve as stable sources of contact and support. In parallel, the stress process model (Pearlin et al., 1990) explains how the chronic stress of caregiving can lead to poor psychological outcomes, including loneliness, while highlighting the protective role of social resources (e.g., interactions and support) in buffering these stressors. Both models highlight the importance of social relationships and their characteristics (e.g., contact, closeness), which may play a unique role in reducing momentary loneliness among dementia caregivers.

Most caregiving studies focus on social contact and support from general social networks, peer support groups, family ties, or a combination of family and friends, often overlooking the distinct role of friendships (Velloze et al., 2022). Indeed, one-third of dementia caregivers from the National Caregivers Training Study mentioned friends in the qualitative interviews, indicating them as a key source of social integration and emotional support (Lilly et al., 2003). Friends may play a particularly important protective role against social isolation and loneliness for dementia caregivers, given the potential loss or deterioration of the relationship with the CR (Adam et al., 2008; Quinn et al., 2009). Moreover, because friendships often involve emotional closeness and warmth (Li et al., 2011), they may, in some cases, be particularly effective in reducing loneliness and are therefore worth examining independently.

For example, studies consistently show that adults with a greater number of friends tend to experience lower levels of loneliness than those with fewer friends (Shiovitz-Ezra & Leitsch, 2010; Thompson et al., 2024), highlighting the importance of friendship quantity. A meta-analysis further found that both the quantity (greater contact) and quality (higher quality) of contact with friends are more strongly associated with reduced loneliness than contact with family among older adults (Pinquart & Sorensen, 2001). However, in the context of dementia caregiving, the emotional impact of interactions may be more complex. Loneliness is not solely determined by whether interactions occur or are positive, but also by whether they meet the caregiver’s emotional needs. For instance, some caregivers may experience guilt or emotional conflict when having fun with friends (Gallego-Alberto et al., 2017; Romero-Moreno et al., 2014), which could reduce the emotional benefits of those positive interactions. That said, drawing on prior studies, we expect that dementia caregivers will generally experience lower momentary loneliness when they have more friends in their social networks, engage in daily interactions with friends, and especially when those interactions are high in quality (e.g., being positive or involving close friends).

Studies have shown bidirectional links between caregiving burden and loneliness, with caregivers who experience greater burden being more vulnerable to increased loneliness, and vice versa (Grycuk et al., 2022; Lee et al., 2017; SuNg et al., 2023). However, limited studies have examined whether social interactions are particularly beneficial in alleviating loneliness for caregivers with higher burden. Compared to dementia caregivers with lower burden, caregivers with higher burden often face increased emotional, physical, and time-related challenges, leading to higher stress and burnout. These challenges may limit their ability to engage socially and seek support (Clark & Bond, 2000; Kotwal et al., 2024), which not only increases their vulnerability to loneliness but also intensifies the emotional significance of each social interaction. Consequently, interactions with friends, which reflect voluntary social engagement and greater pleasantness than other types of social interactions (Ng et al., 2021), may have a particularly strong emotional impact on high-burden caregivers when they occur.

Together, this study addresses three research questions with corresponding hypotheses:

Do dementia caregivers with fewer or no close friends in their network experience more momentary loneliness?

H1: Compared to caregivers with more friends, those with fewer or no close friends will experience higher momentary loneliness, above and beyond demographic factors.

Do interactions with friends temporarily reduce loneliness among dementia caregivers, and does the quality or closeness of these interactions matter?

H2a: Caregivers will experience lower loneliness during periods when they interact with friends compared to periods without such interactions.

H2b: Positive interactions with friends will be associated with momentary reductions in loneliness, whereas negative interactions may exacerbate momentary loneliness.

H2c: Interactions with close friends will be more strongly associated with momentary reductions in loneliness compared to interactions with not close friends.

Does the effect of friend interactions on momentary loneliness vary between high- and low-burden caregivers?

H3: The within-person association between friend interactions and momentary loneliness will be stronger among caregivers with higher caregiving burden.

## Method

### Sample

This study used data from the Stress and Well-Being in the Everyday Lives of Caregivers Study (SWELCare), conducted in the Midwest region of the United States, including Michigan and Ohio, between December 2021 and August 2024 (see the Supplementary Methods section in online supplementary material for detailed recruitment information). Eligible participants were primary caregivers who identified as Black or White, could speak and read English, and cared for someone with signs of dementia—defined as scoring at least two items on the AD8 Dementia Screening Interview, a brief tool for detecting cognitive impairment (Galvin et al., 2005). SWELCare included 247 adults who provided unpaid care to a co-residing adult family member or friend with dementia.

Caregivers completed a 90- to 120-min baseline phone interview covering demographics, caregiving background and conditions, social networks, and self-reported physical and psychological health conditions, followed by a Zoom or phone training session on how to complete mobile surveys. The final analytic sample included 223 caregivers (90%) who participated in both the baseline interview and the 5-day ecological momentary assessment (EMA) study. Caregivers who did not participate in the EMA study (*n *= 24) had higher depression levels than those who did (*t *= 2.34, *p* = .02) but did not differ in demographic, social, or other health characteristics. During the 5-day EMA study, caregivers used a study-provided mobile phone to complete six brief surveys per day, responding to questions about their social interactions and mood.

Caregivers were compensated up to $340, including $50 for the baseline interview, $50 per day for the 5 days of EMA, and a $40 bonus for completing all parts of the study. The study protocol was approved by the University of Michigan Ethics Review Board, and all participants provided informed consent.

### Baseline interview measures

#### Number of close friends in social networks

Caregivers’ close social networks were assessed using the three-circle convoy model (Antonucci et al., 2014), which classifies relationships based on perceived closeness (close, closer, closest). Caregivers could list up to 20 names in each circle. To minimize study fatigue, caregivers provided detailed information, such as the relationship type (e.g., spouse, children, and friends), for each of their closest social partners, capped at 20 social partners (excluding the CR). We created a continuous variable to represent the total number of close friends in the social network (across any circle; Ng et al., 2025). Additionally, in the sensitivity analysis, we tested a binary version of the variable that distinguishes caregivers with at least one close friend from those without, coded as 1 (*with at least one close friend*) and 0 (*without any close friends*).

#### Caregiver burden

Caregiver burden was assessed using the 12-item short version of the Zarit Burden Interview (ZBI-12; Bédard et al., 2001). This measure includes items such as: “*Do you feel that because of the time you spend with the CR, you don’t have enough time for yourself?*” “*Do you feel stressed between caring for CR and trying to meet other responsibilities (work/family)*?” and “*Do you feel that your health has suffered because you are caring for CR?*” The 12 items were rated on a scale from 0 (*Never*) to 4 (*Nearly Always*). Responses were summed to create a total burden score for each participant, ranging from 0 to 48, with higher scores indicating greater caregiver burden. In this study, internal consistency was *α* = 0.88. Following previous literature (Stagg & Larner, 2015), a score of 17 or higher was indicative of high caregiver burden. Based on this threshold, a binary variable was created to classify caregivers into two groups: 1 (*high burden*) and 0 (*low burden*). We also tested this as a continuous moderator in a sensitivity test.

#### Baseline covariates

We adjusted for caregivers’ age (continuous variable), gender (0 =*male*; 1 = *female*), marital status recoded as 0 (*not married*) and 1 (*married/living with partner*), race recoded as 0 (*White*) and 1 (*Black*), education level recoded as 0 (*no college degree*) and 1 (*college degree or above*), working full or part time 0 (*no*) and 1 (*yes*), relationship type with the care recipient 1 (*spouse caregiver*), 2 (*adult child caregiver*), and 3 (*caregiver of other types of relationship*), number of health conditions (e.g., hypertension, diabetes, and cancer), and levels of depression was assessed from 1 (*rarely*) to 4 (*all the* time) using the 8-item CES-D (example items: felt depressed, restless sleep, sad; Gellis, 2010). A sum score from the depression items was generated. We also controlled for the total social network size and the number of close family members (including spouse, children, parents, siblings, grandchildren, and other relatives). We also controlled for years of caregiving and whether the caregiver received help from friends or family members in caring for the CR, to account for the extent of shared caregiving responsibilities. Although many caregiver-related characteristics may capture characteristics of the CR, to balance model complexity, we additionally controlled for key CR’s sociodemographic characteristics, including their age (continuous variable) and gender (0 = *male*; 1 = *female*) (Penning & Wu, 2016; Santoyo-Olsson et al., 2024).

### Ecological momentary assessment measures

The names of individuals in participants’ close social network were preloaded into the EMA surveys and incorporated into specific survey questions. Participants completed EMA surveys on study-provided phones at scheduled times (interval-based measurement): upon waking, 9 a.m., 12 p.m., 3 p.m., 6 p.m., and before bed, over a period of 5 days. Because the waking survey did not include questions about social interactions, it was excluded from the analyses.

#### Social interactions with friends

Caregivers were asked, “*With whom did you interact in the last 3 hours? Interactions can include in person, talking on the phone, through text, email, social media or video chat*,” where they could select preloaded individuals listed in their close social network, as well as an “Other” option. If “Other” was selected, participants were asked, “*What is your relationship with that person?*” and could report up to five individuals not listed in their network. Interactions with friends (both within and outside their close social networks) within each 3-hr interval were coded as 1 (*yes*) or 0 (*no*). Additionally, we created binary variables to indicate any interaction with close friends (i.e., friends who were the top 20 closest social partners; 1 = *yes* or 0 = *no*), and any interaction with non-close friends (i.e., friends not listed as the top 20 closest social partners; 1 = *yes* or 0 = *no*). Consistent with previous research, these variables capture whether any interaction occurs during each 3-hr period, rather than the total amount of time or number of friends interacted with (Ng et al., 2021, 2024).

#### Positive and negative interaction quality with friends

Following the above questions, caregivers were asked: “*Of the interactions you had in the last 3 hours, which ones were positive/enjoyable?*” and “*Of the interactions you had in the last 3 hours, which ones were irritating, hurtful, annoying, or stressful?*” Caregivers indicated which interactions applied to each question. Two binary variables were created: one for positive interactions with friends (1 = *yes* or 0 = *no*) and one for negative interactions with friends (1 = *yes* or 0 = *no*) within each 3-hr interval (Ng et al., 2024). These variables were not mutually exclusive, as caregivers could report both positive and negative interactions or neither within the same 3-hr period.

#### Momentary loneliness

Every 3 hr, participants reported how lonely they felt over the past 3 hr on a scale from 1 (*not at all*) to 5 (*a great deal*).

#### EMA covariates

We adjusted for a comparable set of variables related to interaction with family ties (e.g., any interactions with family, any positive and negative family interactions, any close and non-close family interactions), but we did not aim to directly compare the coefficient or effects of these two social domains. Time of day was dummy-coded into three categories: morning surveys (9 a.m., 12 p.m.) coded as 1 (*yes*) or 0 (*no*), afternoon surveys (3 p.m., 6 p.m.) coded as 1 (*yes*) or 0 (*no*), and before bed surveys coded as 1 (*yes*) or 0 (*no*).

### Analytic strategy

Descriptive statistics and bivariate correlations were calculated at the participant level to explore associations between variables. Intraclass correlation coefficient indicated that 52% of the variance in momentary loneliness [level-1] was due to differences between caregivers, whereas 48% was due to within-person (moment-to-moment) fluctuations. To examine whether the number of close friends in social networks is linked to momentary loneliness, two-level linear models (with assessments [level-1] nested within participants [level-2]) were used, treating momentary loneliness [level-1] as a continuous outcome variable. A continuous variable of the number of close friends was included as a participant-level predictor along with covariates to account for potential confounding effects. This analysis aimed to determine whether the number of close friends uniquely explained variance in momentary loneliness while simultaneously accounting for other key factors.

To examine the daily processes, time-varying predictors (i.e., any interaction with friends, any positive and negative interactions with friends, and any interaction with close and non-close friends) were decomposed into within-person and between-person effects (Curran & Bauer, 2011). Within-person effects (*Level 1*) reflect deviations of an individual assessment’s raw score from the person-level mean, whereas between-person effects (*Level 2*) represent person-specific averages across all assessments within the study period. Analyses and discussion primarily focused on within-person effects because they allow for examining how fluctuations in friendship characteristics are associated with changes in loneliness within the same individual, which allows for stronger inferences about temporal processes that are not captured by between-person comparisons. Momentary loneliness, assessed every 3 hr, was treated as a continuous outcome variable. Participant-level and EMA covariates were controlled, with EMA covariates (e.g., interactions with family) partitioned into within- and between-person effects to capture variance at each level. Models were fitted using restricted maximum likelihood and included random intercepts to account for individual variability.

To examine whether caregiver burden moderates the within-person links between any interaction with friends and momentary loneliness, we included a cross-level interaction term (i.e., any interaction with friends [*Level 1*] × high caregiving burden status [*Level 2*]) in full-sample models. Significant interactions were further explored through simple slope analysis to clarify group-specific associations. All analyses were conducted using Stata 19 MP-2.

## Results

As shown in Table 1, dementia caregivers averaged 61.38 years of age, with 47% aged 65 or older. Most were female (78%), about two-thirds were married and had a college degree or higher, 36% of caregivers identified as Black, and 39% were working full-time/part-time. More than half cared for a spouse, whereas one-third cared for a parent. Caregivers reported an average of two chronic health conditions, moderate depressive symptoms, and 65% were classified as having a high caregiving burden (average burden score of 20.35). On average, they had provided dementia care for 4.63 years (*SD* = 4.25), and 75% received help from friends or family. Caregivers reported an average of 12.53 close ties in their social convoy (*SD *= 8.48, range: 0–47), with 13% listing more than 20 close social ties. Most (78%) had at least one close friend, and nearly all (98%) had a close family member. CRs had a mean age of 75.44 years (*SD* = 11.45), and 50% were female.

**Table 1.** Descriptive statistics (*n *= 223).

Variables	Mean/prop.	*SD*	Min.	Max.
**Caregivers’ baseline characteristics**				
** Age**	61.38	13.19	21.00	87.00
** Female**	0.78			
** Married/cohabitated**	0.64			
** Black**	0.36			
** College degree or above**	0.63			
** Work full-time/part-time**	0.39			
** Relationship types**				
** Spouse/partner caregivers**	0.52			
** Adult child caregivers**	0.36			
** Other caregivers**	0.12			
** # health conditions**	1.92	1.32	0.00	6.00
** Depression**	17.92	3.28	12.00	30.00
** Caregiving burden score**	20.35	8.69	2.00	46.00
** High burden group^a^**	0.65			
** Helping duration (years)**	4.63	4.25	0.08	29.00
** Had help from friends and family members**	0.75			
** Social network size**	12.53	8.48	0.00	47.00
** Number of close friends^b^**	3.23	3.07	0.00	14.00
** Had at least one close friends**	0.78			
** Number of close family^b^**	7.52	4.56	0.00	20.00
** Had at least one close family**	0.98			
**Care recipients’ characteristics**				
** Age**	75.44	11.45	26.00	100.00
** Female**	0.50			
**EMA characteristics**				
** Number of surveys (excluding morning survey)**	21.94	4.87	1.00	40.00
** Momentary loneliness**	1.39	0.63	1.00	5.00
** Any interaction with friends**	0.22	0.22	0.00	0.83
** Any interaction with family**	0.52	0.32	0.00	1.00
** Any positive interaction with friends**	0.19	0.20	0.00	0.83
** Any negative interaction with friends**	0.02	0.04	0.00	0.31
** Any positive interaction with family**	0.47	0.31	0.00	1.00
** Any negative interaction with family**	0.09	0.15	0.00	0.94
** Any interaction with close friends^b^**	0.14	0.19	0.00	0.83
** Any interaction with non-close friends^c^**	0.10	0.14	0.00	0.73
** Any interaction with close family^b^**	0.50	0.33	0.00	1.00
** Any interaction with non-close family^c^**	0.06	0.11	0.00	0.72

*Note*. EMA = ecological momentary assessment.

a A score of 17 or higher on ZBI-12 was indicative of high caregiver burden.

b Close friends or family were the top 20 closest social partners listed in the social convoy.

c Non-close friends or family were individuals not listed as the top 20 closest social partners.

During the 5-day EMA period, caregivers completed an average of 21.94 surveys (*SD *= 4.87, excluding morning surveys). Of these surveys, 22% involved friend interactions; 19% involved positive interactions, 2% involved negative interactions, 14% involved close friends, and 10% involved non-close friends. Among EMA surveys involving friend interactions, 92.2% were purely positive, 3.2% were purely negative, and 4.6% involved both positive and negative elements. See Supplementary Table 1 (see online supplementary material) for participant-level bivariate correlations.

### Number of close friends and momentary loneliness

As shown in Table 2, multilevel linear models accounting for covariates indicated that a greater number of close friends in social networks was associated with reduced momentary loneliness (*B* = −0.03, *p* = .03).

**Table 2.** Multilevel linear models predicting momentary loneliness from the number of close friends in social network.

Variables	*B*		*SE*
**Intercept**	0.68		0.42
**Number of close friends**	−0.03*		0.02
**Participant-level covariates**			
** Age**	0.00		0.00
** Female**	−0.11		0.10
** Married**	−0.32**		0.11
** Black**	−0.12		0.08
** College degree or above**	−0.02		0.07
** Work part-time/full-time**	−0.07		0.07
** Spousal caregivers**		(Ref.)	
** Adult child caregivers**	−0.07		0.16
** Other caregivers**	0.05		0.16
** # health conditions**	−0.06		0.03
** Depression**	0.06***		0.01
** High burden group**	0.35***		0.08
** Help duration in years**	−0.01		0.01
** Help from friends and ­family members**	0.06		0.08
** Social network size**	0.00		0.01
** Number of close family**	−0.01		0.01
** Care recipient age**	−0.00		0.00
** Care recipient female**	−0.04		0.09
**EMA covariates**			
** Morning survey**		(Ref.)	
** Afternoon survey**	−0.02		0.02
** Evening survey**	0.01		0.02

*Note*. EMA = ecological momentary assessment; Ref. = reference. Number of observations = 4,871; number of participants = 223.

* *p* < .05.

** *p* < .01.

*** *p* < .001.

Although not the primary focus, caregivers who were unmarried (*B *= −0.32, *p* = .004), higher levels of depression (*B *= 0.06, *p* < .001) and with higher caregiving burden experienced higher levels of momentary loneliness (*B *= 0.34, *p* < .001) compared to those who were married, lower levels of depression, and with lower caregiving burden. The number of close family members was not associated with momentary loneliness (*B* = −0.01, *p* = .42).

### Links between daily friend interactions and momentary loneliness

As shown in Table 3, Model 1, we first tested the within-person links between friend interactions and momentary loneliness. Multilevel linear models revealed that caregivers reported lower levels of loneliness during times when they interacted with friends compared to when they did not (*B *= −0.06, *p* = .007).

**Table 3.** Multilevel linear models predicting momentary loneliness from friend interactions.

Variables	Model 1			Model 2			Model 3		
	*B*		*SE*	*B*		*SE*	*B*		*SE*
**Intercept**	0.73		0.42	0.79		0.42	0.73		0.41
**Within-person effects**									
** Friend interactions**	−0.06**		0.02	–		–	–		–
** Positive friend interactions**	–		–	−0.07**		0.02	–		–
** Negative friend interactions**	–		–	0.08		0.07	–		–
** Close friend interactions**	–		–	–		–	−0.05		0.03
** Non-close friend interactions**	–		–	–		–	−0.08*		0.03
**Between-person effects**									
** Friend interactions**	0.18		0.16	–		–	–		–
** Positive friend interactions**	–		–	0.04		0.18	–		–
** Negative friend interactions**	–		–	1.43		0.88	–		–
** Close friend interactions**	–		–	–		–	−0.05		0.19
** Non-close friend interactions**	–		–	–		–	0.49		0.25
**Participant-level covariates**									
** Age**	0.00		0.00	0.00		0.00	−0.00		0.00
** Female**	−0.15		0.10	−0.12		0.10	−0.12		0.10
** Married**	−0.31**		0.12	−0.31**		0.12	−0.33**		0.12
** Black**	−0.13		0.08	−0.14		0.08	−0.14		0.08
** College degree or above**	−0.05		0.07	−0.05		0.07	−0.05		0.07
** Work part-time/full-time**	−0.06		0.07	−0.06		0.07	−0.06		0.07
** Spousal caregivers**		(Ref.)			(Ref.)			(Ref.)	
** Adult child caregivers**	−0.06		0.17	−0.07		0.16	−0.09		0.16
** Other caregivers**	0.02		0.16	0.02		0.16	−0.00		0.16
** # health conditions**	−0.05		0.03	−0.06*		0.03	−0.05		0.03
** Depression**	0.06***		0.01	0.06***		0.01	0.06***		0.01
** High burden group**	0.31***		0.08	0.29***		0.08	0.31***		0.08
** Social network size**	−0.01*		0.00	−0.01		0.00	−0.01*		0.00
** Help duration in years**	−0.01		0.01	−0.01		0.01	−0.00		0.01
** Help from friends and family members**	0.03		0.08	0.03		0.08	0.04		0.08
** Care recipient age**	−0.00		0.00	−0.00		0.00	−0.00		0.00
** Care recipient female**	−0.06		0.09	−0.06		0.09	−0.03		0.09
**EMA covariates**									
** Morning survey**		(Ref.)			(Ref.)			(Ref.)	
** Afternoon survey**	−0.03		0.02	−0.03		0.02	−0.03		0.02
** Evening survey**	−0.01		0.02	−0.00		0.02	−0.01		0.02
**Within-person effects**									
** Family interactions**	−0.02		0.02	–		–	–		–
** Positive family interactions**	–		–	−0.06**		0.02	–		–
** Negative family interactions**	–		–	0.08*		0.03	–		–
** Close family interactions**	–		–	–		–	−0.01		0.02
** Non-close family interactions**	–		–	–		–	−0.13**		0.04
**Between-person effects**									
** Family interactions**	−0.03		0.12	–		–	–		–
** Positive family interactions**	–		–	−0.08		0.13	–		–
** Negative family interactions**	–		–	0.20		0.25	–		–
** Close family interactions**	–		–	–		–	−0.01		0.12
** Non-close family interactions**	–		–	–		–	−0.01		0.30

*Note*. EMA = ecological momentary assessment; Ref. = reference. Number of observations = 4,357; number of participants = 223.

* *p* < .05.

** *p* < .01.

*** *p* < .001.

Next, we tested whether the quality of interactions with friends (positive and negative interactions with friends as separate predictors) was linked to momentary loneliness. Multilevel linear models revealed that caregivers reported lower levels of loneliness during times when they had positive interactions with friends compared to when they did not (*B *= −0.07, *p* = .005; Table 3, Model 2). In contrast, negative interactions with friends were not associated with temporary changes in loneliness (*B *= 0.08, *p* = .29).

Furthermore, we tested whether the closeness of friends matters (interactions with close and non-close friends were treated as two separate predictors). Multilevel linear models revealed that caregivers reported lower levels of loneliness during times when they interacted with non-close friends compared to when they did not (*B *= −0.08, *p* = .01; Table 3, Model 3). However, interactions with close friends were only marginally linked to lower loneliness (*B *= −0.05, *p* = .06).

Notably, all of these findings accounted for family factors, suggesting the robustness of the results. Although this was not central to our investigation, as shown in Table 3, positive family interactions were associated with momentary reductions in loneliness (*B *= −0.06, *p* = .004), whereas negative family interactions were linked to increases in loneliness (*B *= 0.08, *p* = .03). Similarly, non-close family interactions were also associated with reduced loneliness (*B *= −0.13, *p* = .001).

### Moderating effects of caregiver burden

**Figure 1.**
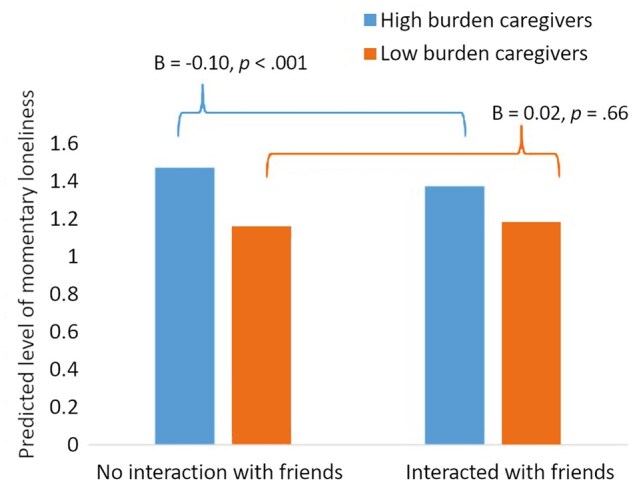
The within-person effect of friend interactions on momentary loneliness by caregiving burden.

We tested whether the within-person association between interactions with friends and momentary loneliness differed between high- and low-burden caregivers. A significant interaction was found between friend interactions × high caregiving burden group (*B *= −0.12, *p* = .02; Table 4). Simple slope analyses indicated that for high-burden caregivers, interacting with friends was associated with lower momentary loneliness (*B *= −0.10, *p* < .001). However, this association was nonsignificant for low-burden caregivers (*B *= 0.02, *p* = .66; see Figure 1).

**Table 4.** Multilevel linear models predicting momentary loneliness from interactions with friends: moderating roles of caregiving burden.

Variables	*B*		*SE*
**Intercept**	0.67		0.43
**High burden group**	0.45**		0.16
**Within-person effects**			
** Friend interactions**	0.02		0.04
** Friend interactions × high burden group**	−0.12*		0.05
**Between-person effects**			
** Friend interactions**	0.37		0.27
** Friend interactions × high burden group**	−0.30		0.33
**Participant-level covariates**			
** Age**	−0.00		0.00
** Female**	−0.15		0.10
** Married**	−0.31**		0.12
** Black**	−0.14		0.08
** College degree or above**	−0.05		0.07
** Work part-time/full-time**	−0.06		0.07
** Spousal caregivers**	(REF.)		(REF.)
** Adult child caregivers**	−0.08		0.17
** Other caregivers**	0.01		0.16
** # health conditions**	−0.04		0.03
** Depression**	0.06***		0.01
** Social network size**	−0.01*		0.00
** Help duration in years**	−0.01		0.01
** Help from friends and family members**	0.03		0.08
** Care recipient age**	−0.00		0.00
** Care recipient female**	−0.05		0.09
**EMA covariates**			
** Morning survey**	(REF.)		(REF.)
** Afternoon survey**	−0.03		0.02
** Evening survey**	−0.01		0.02
** Within-person effects**			
** Family interactions**	0.00		0.04
** Family interactions × high burden group**	−0.04		0.05
** Between-person effects**			
** Family interactions**	0.07		0.19
** Family interactions × high burden group**	−0.13		0.22

*Note*. EMA = ecological momentary assessment. Number of observations = 4,357. Number of participants = 223.

* *p* < .05.

** *p* < .01.

*** *p* < .001.

A series of sensitivity tests were conducted to ensure the robustness of the findings. These included: (a) reestimating models without family controls; (b) using a binary close friend variable instead of a continuous one; (c) testing for curvilinear effects of close friend number; (d) examining reverse and lagged associations; (e) using categorical social context (alone, with friends only, with others only [excluding CR], with both friends and others [excluding CR]) as predictors; (f) testing how the extent of being alone was associated with momentary loneliness; (g) testing caregiving burden as a continuous moderator; (h) examining whether associations between friend interaction quality (positive vs negative) or closeness (close vs non-close) and loneliness differed by burden groups in separate models; and (i) tested a three-way interaction. Findings can be found in Supplementary Tables 2–7 and Supplementary Figures 1 and 2 (see online supplementary material).

## Discussion

Addressing loneliness in dementia caregivers is crucial, as it affects their health, mortality, as well as the quality of care they provide (Hajek et al., 2021; Holt-Lunstad, 2024). This study is among the first to examine daily fluctuations in loneliness among dementia caregivers. As research increasingly prioritizes friendships alongside or even above family ties, understanding their role in caregiving becomes crucial (Blieszner et al., 2019). Beyond simply assessing whether social interactions with friends occur, this study examines how the quality of these interactions (positive vs. negative) and the closeness of friendships (close vs not close friends) influence loneliness, furthering our understanding of how different aspects of friend interactions contribute to caregivers’ momentary loneliness.

For the first research question, each additional close friend was linked to a 0.03-unit reduction in loneliness among dementia caregivers. Although recent research suggests that the effect of additional close friends on loneliness may diminish once a threshold of four close friends is reached (Thompson et al., 2024), findings from the sensitivity tests did not show evidence of such a curvilinear relationship. Notably, the link between friendship networks and loneliness varied depending on how friendship was operationalized. When friendship was treated as a binary variable (i.e., whether or not caregivers had close friends in their networks), caregivers with or without close friends reported similar levels of momentary loneliness. This contradicts previous studies that have found that adults without friends experience higher levels of loneliness than those with at least one friend (Savikko et al., 2005) and suggests that simply having close friends may not be sufficient to reduce loneliness in the caregiving context. Instead, the quantity of close friends and the frequency of daily contact with them may play a more significant role in alleviating loneliness.

Although not the primary focus, findings showed that caregivers with higher burden experience greater momentary loneliness compared to those with lower burden, indicating that caregiving stress may contribute to loneliness (Victor et al., 2021), which aligns with the stress process model (Pearlin et al., 1990). Consistent with prior caregiving research (Beeson, 2003), we found that depression was strongly associated with momentary loneliness in dementia caregivers. Notably, loneliness may be both a cause and a consequence of depression, but studies have indicated that loneliness, rather than depression, is a likely point of intervention to improve caregiver well-being (Holt-Lunstad, 2024; Peavy et al., 2022). None of the other social factors (number of close family or overall social network size) was significantly associated with momentary loneliness, except being married.

The second research question bridges global measures of close friend networks with momentary social interactions involving both close and non-close friends in real-life settings. Findings revealed that caregivers experienced lower loneliness (a 0.06-unit reduction) during times when they interacted with friends compared to times without such interactions. This supports our hypothesis that engaging with friends offers immediate, albeit potentially temporary, relief from feeling lonely. This is consistent with the broader literature, which shows that social interactions with friends are linked to enhanced mood in both the general adult and older populations (Ng et al., 2021, 2023).

In addition to the mere occurrence of friend interactions, the qualitative aspects of these interactions emerged as a crucial factor. Our findings showed that reductions in loneliness were driven by positive interactions with friends, whereas negative interactions with friends showed no significant association with momentary loneliness. Positive interactions may include experiences such as feeling supported, enjoying companionship, or having meaningful conversations. In contrast, negative interactions may involve conflict, feeling ignored/annoyed or experiencing tension or disappointment. This finding lends support to our hypothesis and suggests that friend interactions contribute to well-being primarily through their positive nature (Ng et al., 2021, 2025). Although prior research has indicated that negative interactions with social partners can be particularly salient to health outcomes, including loneliness (Umberson & Montez, 2010), negative interactions with friends were infrequent in this study, occurring in only 2% of all EMA surveys, and did not exacerbate loneliness among caregivers. This could be because caregivers tend to selectively retain supportive friends while distancing themselves from negative ones over time (Charles & Carstensen, 2010), or, as suggested by the social input model (Fingerman & Charles, 2010), friends may minimize conflicts or tensions to maintain positive emotional experiences, which are crucial for the caregiver’s emotional well-being and sense of connection.

Interestingly, and contrary to prevailing theoretical models of social convoy (Antonucci et al., 2014), interactions with non-close friends were more strongly linked to reductions in loneliness than interactions with close friends. This finding challenges the assumption that close friendships provide the most substantial emotional benefits and suggests that weaker ties may serve as an effective avenue for alleviating loneliness among older adults (Lam et al., 2023). One possible explanation is that interactions with non-close friends may be more likely to take place outside the home, which has been associated with lower momentary loneliness (Compernolle et al., 2021; Goldman & Compernolle, 2023). Additionally, non-close friends may offer novel experiences, social variety, and access to new social circles, which can provide a refreshing and uplifting emotional boost (Ng et al., 2021). Alternatively, because caregiving demands can strain close relationships (e.g., create conflicts), weaker ties, being more malleable and flexible, may play a compensatory role in helping caregivers maintain social engagement despite diminished interactions with close ties (Huxhold et al., 2020; Lam et al., 2023). These findings suggest the potential of fostering weak and diverse social interactions as a strategy for mitigating loneliness among caregiver populations.

Regarding the final research question, our findings support the hypothesis that the emotional benefit of friend interactions in reducing loneliness is more pronounced for caregivers with higher burden. Sensitivity tests further showed that only high-burden caregivers experienced reduced loneliness from positive friend interactions and interactions with non-close friends. This aligns with the stress-buffering model (Cohen & Wills, 1985), which suggests that social interactions are particularly beneficial for those experiencing higher levels of stress. For caregivers with higher burden, interactions with friends may offer a temporary respite, providing emotional support, companionship, and a sense of normalcy amid caregiving demands (Lilly et al., 2003). In contrast, caregivers with lower burden may not experience as significant a reduction in loneliness from friend interactions, as their overall loneliness and need for support may be relatively lower. By comparing daily experiences of high- versus low-burden caregivers, this study helps identify those most at risk for loneliness.

Although our primary focus is on friendships, the significant associations between family interactions and momentary loneliness highlight the complementary roles that both friendship and family ties play in shaping momentary feelings of loneliness and suggest that interventions aiming to reduce loneliness may consider the broader social context, addressing both friendships and family ties. Moreover, the significant within-person effects alongside the nonsignificant between-person effects may reflect the immediate, contextual benefit of friend interactions in providing temporary relief from loneliness, whereas more stable traits or circumstances (e.g., marital status, depression) may better explain between-person differences in loneliness.

### Limitations and future directions

Despite its contributions, this study has several limitations that warrant consideration. First, this study focused on friend interactions and did not examine their role in providing or assisting with dementia caregiving; future research could explore this potential role of friends. Although EMA offers a dynamic and ecologically valid measure of social interactions and loneliness, the 5-day observation period may not be able to capture variability in social experiences and longer-term patterns, as some caregivers may interact with friends only biweekly or monthly. Future research could extend the study duration or incorporate longitudinal designs to examine how friend interactions influence loneliness over time. Similarly, as with all self-reported data, the potential for social desirability bias cannot be entirely ruled out, highlighting the need for more objective data collection methods (e.g., call/social media records (Ng, 2020, 2023) and electronically activated recordings, and physiological measures for mood) in future studies. Finally, the unique challenges of dementia caregiving suggest that the patterns observed in this study may not fully generalize to dementia caregivers in other countries or to caregivers of individuals without dementia. Future research could conduct cross-national studies (Ng, 2025) or compare these findings with other caregiver populations.

In sum, this study examined friend interactions and momentary loneliness among dementia caregivers using innovative EMA methodology, providing valuable insights into the social determinants of loneliness and informing the design of targeted interventions to help reduce loneliness in this population. For example, friendship-focused behavioral activation strategies could encourage caregivers, especially those with higher burden to intentionally engage with friends through accessible activities integrated into their daily routines, such as regular phone calls, virtual meetups, or shared hobbies. Additionally, interventions could support caregivers in managing caregiving demands that limit social time and in fostering supportive friend networks. These approaches, based on the findings, might provide practical solutions to alleviate loneliness and enhance overall caregiver health.

## Supplementary Material

gbaf190_Supplementary_Data

## Data Availability

Data, analytic methods, and materials are available to other researchers upon request. The current study reported in this manuscript was not preregistered.
