# Supplementary material for: Friendship and momentary loneliness in dementia caregiving: daily experiences of caregivers with high and low burden
Source: J Gerontol B Psychol Sci Soc Sci. 2025 Oct 1;80(12):gbaf190. doi: 10.1093/geronb/gbaf190 (PMC12596082; doi:10.1093/geronb/gbaf190)

***The Journals of Gerontology, Series B: Psychological Sciences and Social Sciences* Supplementary Material: Ng, Turkelson, Kratz, & Birditt. Friendship and Momentary Loneliness in Dementia Caregiving: Daily Experiences of Caregivers with High and Low Burden.**

**Supplementary Methods**

**Study Participants Recruitment**

SWELcare participants were recruited from Michigan (southeastern Detroit, West, Central, Thumb, Traverse City areas) and Ohio (areas near the Michigan border, Columbus, Cincinnati, and Dayton areas). Recruitment sites included: the University of Michigan Alzheimer’s Disease Research Center (MADRC) recruitment site (http://alzheimers.med.umich.edu/research); the Wayne State University Healthier Black Elders Center (HBEC); University of Michigan Health Research Portal (https://umhealthresearch.org); the Area Agency on Aging; the Community Aging Network; local community organizations and public spaces (libraries, grocery stores, etc.) Participants were directly recruited from names provided by MADRC and HBEC or who expressed interest through the UM Health Research Portal and the study website. Other methods included contacting groups, centers, businesses, public spaces and asking them to post the study’s recruitment flyers, which led to participants contacting the study phone and being screened for eligibility.

**Post Hoc tests**

A series of sensitivity tests were conducted to ensure the robustness of the findings. Findings were:

1. We estimated all models without family controls, and the patterns of findings remained unchanged.
2. We examined a binary variable representing any close friends in a social network instead of using a continuous variable for RQ1. Having close friends was not associated with momentary loneliness (*B* = 0.05, *p* = .59).
3. We tested the potential curvilinear relationship between the number of close friends and momentary loneliness in dementia caregivers to assess whether there is a limit to the benefits of having more friends by including a quadratic term (the square of the number of friends). Findings revealed the quadratic term was non-significant (*B* = -0.002, *p* = 0.59), indicating that the linear model adequately captures the association between the number of close friends and momentary loneliness.
4. We tested the reverse association. Multilevel logistic models showed that higher-than-usual momentary loneliness was linked to less likelihood of interaction with friends in the same 3-hr period (*Odd Ratio* [*OR*] =0.81, *p*=.007; **Supplementary Table 2**). We also conducted a lagged analysis to examine whether interactions with friends were associated with reduced loneliness at the subsequent assessment. Multilevel linear models indicated that within-person interactions with friends were linked to lower momentary loneliness at the next time point (*B* = -0.09, *p* = .001; see **Supplementary Table 3**).
5. EMA studies have found that adults experienced higher levels of loneliness when they were alone compared to being with others (older adults: Compernolle et al., 2021; Zhaoyang et al., 2022; young adults sample: Rodriguez et al., 2025). Thus, we also explored using a categorical variable representing social context: (1) alone, (2) with friends only, (3) with others only (not including the person living with dementia), and (4) with both friends and others (not including the person living with dementia) as a predictor of momentary loneliness. Results showed that, compared to periods when caregivers were alone, interactions with friends only were associated with reduced momentary loneliness (*B* = 0.10, *p* = .04). Yet, compared to being alone, interactions with others (B = -0.001, p = .97) and interactions with both friends and others (B = 0.03, p = .45) did not differ in momentary loneliness (see Supplementary Table 4).
6. We also examined whether the amount of time spent alone (measured in hours) was associated with momentary loneliness among dementia caregivers. Multilevel linear models revealed that spending greater-than-usual time alone was significantly associated with higher levels of momentary loneliness (*B* = 0.03, *p* = .002; **Supplementary Table 5**).
7. Instead of a binary variable (high versus low caregiving burden), we also tested caregiving burden as a continuous moderator, but the interaction term between friend interactions and continuous version of caregiving burden on loneliness was non-significant (*B* = -0.01, *p* = .10).
8. We also examined whether the links between positive and negative friend interactions, as well as close and non-close friend interactions, and momentary loneliness varied between high-burden vs low-burden caregivers in two separate models. Findings revealed that the within-person effects of positive friend interactions and non-close friend interactions on reduced momentary loneliness were observed only among high-burden caregivers (see **Supplementary Table 6-7 and Supplementary Figure 1-2**).
9. Finally, we also tested a three-way interaction (friend interactions [*level 1*] × burden group [*level 2*] × number of close friends [*level 2*]) to examine whether friends reduce loneliness most for high-burden caregivers with more close friends. However, the three-way interaction was non-significant (*B* = 0.01, *p* = .53).

**Supplementary Table 1.** *Bivariate Corrections*

|  | 1 | 2 | 3 | 4 | 5 | 6 | 7 | 8 | 9 | 10 | 11 | 12 | 13 | 14 | 15 | 16 | 17 | 18 | 19 | 20 | 21 | 22 | 23 | 24 | 25 | 26 | 27 | 28 | 29 | 30 |
| --- | --- | --- | --- | --- | --- | --- | --- | --- | --- | --- | --- | --- | --- | --- | --- | --- | --- | --- | --- | --- | --- | --- | --- | --- | --- | --- | --- | --- | --- | --- |
| 1. Age | - |  |  |  |  |  |  |  |  |  |  |  |  |  |  |  |  |  |  |  |  |  |  |  |  |  |  |  |  |  |
| 2. Female | -.14* | - |  |  |  |  |  |  |  |  |  |  |  |  |  |  |  |  |  |  |  |  |  |  |  |  |  |  |  |  |
| 3. Married/Cohabitated | .39  *** | -.09 | - |  |  |  |  |  |  |  |  |  |  |  |  |  |  |  |  |  |  |  |  |  |  |  |  |  |  |  |
| 4. Non-Hispanic Black | -.08 | .09 | -.36  *** | - |  |  |  |  |  |  |  |  |  |  |  |  |  |  |  |  |  |  |  |  |  |  |  |  |  |  |
| 5. College degree+ | .14* | -.04 | .07 | -.06 | - |  |  |  |  |  |  |  |  |  |  |  |  |  |  |  |  |  |  |  |  |  |  |  |  |  |
| 6. Work part-time/full-time | -.29  *** | -.01 | -.06 | .01 | .08 | - |  |  |  |  |  |  |  |  |  |  |  |  |  |  |  |  |  |  |  |  |  |  |  |  |
| 7. Spouse caregivers | .56  *** | -.11 | .78  *** | -.39  *** | .07 | -.14* | - |  |  |  |  |  |  |  |  |  |  |  |  |  |  |  |  |  |  |  |  |  |  |  |
| 8. Adult child caregivers | -.41  *** | .09 | -.61  *** | .32  *** | .00 | .08 | -.78  *** | - |  |  |  |  |  |  |  |  |  |  |  |  |  |  |  |  |  |  |  |  |  |  |
| 9. Other caregivers | -.27  *** | .03 | -.29  *** | .12 | -.11 | .10 | -.38  *** | -.28  *** | - |  |  |  |  |  |  |  |  |  |  |  |  |  |  |  |  |  |  |  |  |  |
| 10. # health conditions | .37  *** | .01 | .13 | .02 | -.12 | -.19  ** | .26  *** | -.21  ** | -.08 | - |  |  |  |  |  |  |  |  |  |  |  |  |  |  |  |  |  |  |  |  |
| 11. Depression | -.34  *** | .08 | -.15* | .09 | -.04 | .04 | -.15* | .16* | -.01 | -.05 | - |  |  |  |  |  |  |  |  |  |  |  |  |  |  |  |  |  |  |  |
| 12. High burden group | -.14* | .17* | -.03 | -.13 | .14* | .05 | -.05 | .16* | -.16* | -.04 | .21** | - |  |  |  |  |  |  |  |  |  |  |  |  |  |  |  |  |  |  |
| 13. Social network size | .32  *** | .10 | .34  *** | -.17  ** | .12 | -.07 | .38  *** | -.26  *** | -.19  ** | .07 | -.19  ** | -.12 | - |  |  |  |  |  |  |  |  |  |  |  |  |  |  |  |  |  |
| 14. Any close friends | .03 | .28  *** | -.03 | .00 | .25  *** | .01 | .02 | .05 | -.10 | -.09 | .06 | .15* | .24  *** | - |  |  |  |  |  |  |  |  |  |  |  |  |  |  |  |  |
| 15. Any close family | .12 | .01 | .11 | .10 | -.03 | .04 | .07 | -.11 | .05 | .02 | -.07 | -.10 | .15* | .17* | - |  |  |  |  |  |  |  |  |  |  |  |  |  |  |  |
| 16. Help duration | .06 | .02 | -.07 | .05 | .02 | -.00 | -.06 | .10 | -.06 | .11 | .06 | .12 | -.02 | .03 | -.02 | - |  |  |  |  |  |  |  |  |  |  |  |  |  |  |
| 17. Help from friends/family | .01 | -.08 | -.04 | -.00 | .12 | .03 | -.08 | .04 | .05 | -.08 | -.12 | -.03 | .14* | .09 | .00 | -.09 | - |  |  |  |  |  |  |  |  |  |  |  |  |  |
| 18. Care recipient age | .31  *** | .09 | -.25  *** | .21  ** | .12 | -.11 | -.32  *** | .42  *** | -.13 | -.02 | -.15* | .10 | .03 | .08 | -.07 | .11 | .08 | - |  |  |  |  |  |  |  |  |  |  |  |  |
| 19. Care recipient female | -.13 | -.41  *** | -.40  *** | .20  ** | .04 | .03 | -.45  *** | .48  *** | -.01 | -.06 | -.03 | -.10 | -.23  *** | -.13* | -.00 | .01 | .11 | .18  ** | - |  |  |  |  |  |  |  |  |  |  |  |
| 20. Number of surveys | .06 | .02 | .22  *** | -.23  *** | .00 | -.04 | .18  ** | -.13 | -.09 | .00 | -.08 | .01 | .20  ** | .07 | -.02 | .01 | -.05 | -.05 | -.19  ** |  |  |  |  |  |  |  |  |  |  |  |
| 21. Momentary loneliness | -.25  *** | -.01 | -.25  *** | -.03 | -.01 | .00 | -.19  ** | .15* | .08 | -.17* | .42  *** | .32  *** | -.26  *** | .04 | -.08 | -.02 | -.00 | -.04 | .04 | -.15* | - |  |  |  |  |  |  |  |  |  |
| 22. Friend interaction | .07 | .14* | -.10 | .00 | .23  *** | .02 | -.07 | .03 | .06 | -.07 | -.11 | .01 | .14* | .34  *** | .02 | .15* | .01 | .15* | -.05 | -.02 | -.00 | - |  |  |  |  |  |  |  |  |
| 23. Family interaction | -.22  ** | .22  *** | .04 | .10 | .02 | .05 | -.21  ** | .20  ** | .02 | -.19  ** | -.00 | -.06 | .11 | .07 | .11 | -.06 | .11 | .02 | -.02 | .02 | -.04 | .03 | - |  |  |  |  |  |  |  |
| 24. Positive friend interaction | .07 | .14* | -.05 | -.02 | .19  ** | .03 | -.02 | -.02 | .06 | -.07 | -.09 | .01 | .15* | .33  *** | .01 | .17* | .02 | .12 | -.05 | -.03 | -.01 | .96  *** | .03 | - |  |  |  |  |  |  |
| 25. Negative friend interaction | -.07 | -.08 | -.16* | .09 | .12 | .01 | -.13* | .12 | .03 | .11 | .15* | .12 | -.12 | .12 | .05 | .20  ** | .00 | .04 | .07 | .03 | .17* | .39  *** | -.03 | .31  *** | - |  |  |  |  |  |
| 26. Positive family interaction | -.21  ** | .23  *** | .06 | .05 | .04 | .04 | -.18  ** | .17* | .03 | -.18  ** | -.03 | -.08 | .11 | .09 | .11 | -.07 | .13 | .01 | -.04 | .04 | -.06 | .03 | .96  *** | .04 | -.04 | - |  |  |  |  |
| 27. Negative family interaction | -.29  *** | .15* | .04 | .06 | -.04 | .09 | -.17  ** | .16* | .03 | -.14* | .22  *** | .15* | -.03 | -.03 | .06 | -.09 | .02 | -.07 | -.01 | -.01 | .11 | -.06 | .47  *** | -.06 | -.05 | .38  *** | - |  |  |  |
| 28. Close friend interaction | -.01 | .18  ** | -.16* | -.03 | .19  ** | .05 | -.11 | .07 | .06 | -.09 | -.06 | .05 | .12 | .41  *** | .00 | .17* | .07 | .13 | -.01 | -.05 | -.01 | .84  *** | .04 | .84  *** | .31  *** | .04 | -.05 | - |  |  |
| 29. Non-close friend interaction | .20  ** | .01 | .06 | .01 | .14* | -.08 | .07 | -.09 | .02 | .01 | -.12 | -.04 | .14* | .06 | .04 | .03 | -.06 | .12 | -.14* | .06 | .01 | .65  *** | -.04 | .58  *** | .29  *** | -.05 | -.08 | .17  ** | - |  |
| 30. Close family interaction | -.23  *** | .25  *** | .05 | .09 | .00 | .05 | -.21  ** | .20  ** | .02 | -.17* | .00 | -.04 | .12 | .08 | .10 | -.06 | .11 | .03 | -.03 | .01 | -.04 | .03 | .98  *** | .02 | -.02 | .94  *** | .48  *** | .04 | -.05 | - |
| 31. Non-close family interaction | .02 | -.09 | -.09 | .02 | .11 | .06 | -.05 | .06 | -.00 | -.08 | -.06 | -.04 | -.03 | .04 | .07 | -.02 | .05 | .02 | .08 | -.02 | .02 | .20** | .17* | .18** | .08 | .15* | -.06 | .14* | .16* | .04 |

**p* <.05; ***p* <.01; *** *p*<.001

**Supplementary Table 2.** *Multilevel Logistic Models Predicting Friend Interactions from Momentary Loneliness*

|  | *B* | *Odd Ratio* |  | *SE* |
| --- | --- | --- | --- | --- |
| Intercept | -3.13 | 0.04 | * | 1.40 |
| **Within-person effects** |  |  |  |  |
| Momentary loneliness | -0.22 | 0.81 | ** | 0.08 |
| **Between-person effects** |  |  |  |  |
| Momentary loneliness | 0.27 | 1.31 |  | 0.22 |
| Covariates |  |  |  |  |

Notes. Number of observations = 4,357. Number of participants = 223. Covariates include caregivers’ age, gender, marital status, race, education, work status, relationship with care recipient, health condition, depression symptoms, burden group, years of helping, any help from family and friends, social network size, care recipient’s age and gender, and time of day.

**p* <.05; ***p* <.01.

**Supplementary Table 3.** *Multilevel Linear Models Predicting Momentary Loneliness at the subsequent assessment from Friend Interactions*

|  | *B* |  | *SE* |
| --- | --- | --- | --- |
| Intercept | 0.97 | * | 0.41 |
| **Within-person effects** |  |  |  |
| Friend interactions | -0.09 | ** | 0.03 |
| **Between-person effects** |  |  |  |
| Friend interactions | 0.22 |  | 0.16 |
| Covariates |  |  |  |

Notes. Number of observations =3,335. Number of participants = 220. Covariates include caregivers’ age, gender, marital status, race, education, work status, relationship with care recipient, health condition, depression symptoms, burden group, years of helping, any help from family and friends, social network size, care recipient’s age and gender, time of day, and family interactions.

**p* <.05; ***p* <.01.

**Supplementary Table 4.** *Multilevel Linear Models Predicting Momentary Loneliness from Different Social Contexts*

|  | *B* |  | *SE* |
| --- | --- | --- | --- |
| Intercept | 0.72 |  | (0.42) |
| Alone |  | (REF.) |  |
| Friend interactions | -0.10 | * | (0.05) |
| Other interactions (not including person living with dementia) | -0.00 |  | (0.03) |
| Both friend and other interactions (not including person living with dementia) | 0.03 |  | (0.03) |
| Covariates |  |  |  |

Notes. Number of observations = 4,357. Number of participants = 223. Covariates include caregivers’ age, gender, marital status, race, education, work status, relationship with care recipient, health condition, depression symptoms, burden group, years of helping, any help from family and friends, social network size, care recipient’s age and gender, and time of day.

**p* <.05.

**Supplementary Table 5.** *Multilevel Linear Models Predicting Momentary Loneliness from the Amount of Time Spent Alone (Measured in Hours)*

|  | *B* |  | *SE* |
| --- | --- | --- | --- |
| Intercept | 0.66 |  | 0.42 |
| **Within-person effects** |  |  |  |
| Time spent alone | 0.03 | ** | 0.01 |
| **Between-person effects** |  |  |  |
| Time spent alone | 0.11 |  | 0.06 |
| Covariates |  |  |  |

Notes. Number of observations = 4,357. Number of participants = 223. Covariates include caregivers’ age, gender, marital status, race, education, work status, relationship with care recipient, health condition, depression symptoms, burden group, years of helping, any help from family and friends, social network size, care recipient’s age and gender, and time of day.

***p* <.01

**Supplementary Table 6.** *Multilevel Linear Models Predicting Momentary Loneliness from the Quality of Friend Interactions: Moderating Roles of Caregiving Burden*

|  | *B* |  | *SE* |
| --- | --- | --- | --- |
| Intercept | 0.72 |  | 0.42 |
| High burden group | 0.42 | ** | 0.15 |
| **Within-person effects** |  |  |  |
| Positive friend interactions | 0.03 |  | 0.04 |
| Positive friend interactions × High burden group | -0.14 | ** | 0.05 |
| Negative friend interactions | -0.10 |  | 0.17 |
| Negative friend interactions × High burden group | 0.21 |  | 0.19 |
| **Between-person effects** |  |  |  |
| Positive friend interactions | 0.32 |  | 0.28 |
| Positive friend interactions × High burden group | -0.52 |  | 0.36 |
| Negative friend interactions | -0.23 |  | 1.84 |
| Negative friend interactions × High burden group | 2.41 |  | 2.05 |
| Covariates |  |  |  |

Note: Number of observations = 4,357. Number of participants = 223. Covariates include caregivers’ age, gender, marital status, race, education, work status, relationship with care recipient, health condition, depression symptoms, burden group, years of helping, any help from family and friends, social network size, care recipient’s age and gender, time of day, and positive and negative family interactions.

***p* <.01.

**Supplementary Table 7.** *Multilevel Linear Models Predicting Momentary Loneliness from the Closeness of Friend Interactions: Moderating Roles of Caregiving Burden*

|  | *B* |  | *SE* |
| --- | --- | --- | --- |
| Intercept | 0.63 |  | 0.42 |
| High burden group | 0.48 | ** | 0.15 |
| **Within-person effects** |  |  |  |
| Close friend interactions | -0.04 |  | 0.05 |
| Close friend interactions × High burden group | -0.03 |  | 0.06 |
| Non-close friend interactions | 0.06 |  | 0.05 |
| Non-close friend interactions × High burden group | -0.21 | ** | 0.07 |
| **Between-person effects** |  |  |  |
| Close friend interactions | 0.03 |  | 0.32 |
| Close friend interactions × High burden group | -0.12 |  | 0.39 |
| Non-close friend interactions | 0.77 | * | 0.38 |
| Non-close friend interactions × High burden group | -0.49 |  | 0.51 |
| Covariates |  |  |  |

Note: Number of observations = 4,357. Number of participants = 223. Covariates include caregivers’ age, gender, marital status, race, education, work status, relationship with care recipient, health condition, depression symptoms, burden group, years of helping, any help from family and friends, social network size, care recipient’s age and gender, time of day, and close and non-close family interactions.

**p* <.05; ***p* <.01.

**Supplementary Figure 1.** *The within-person effect of positive friend interactions on momentary loneliness by caregiving burden*


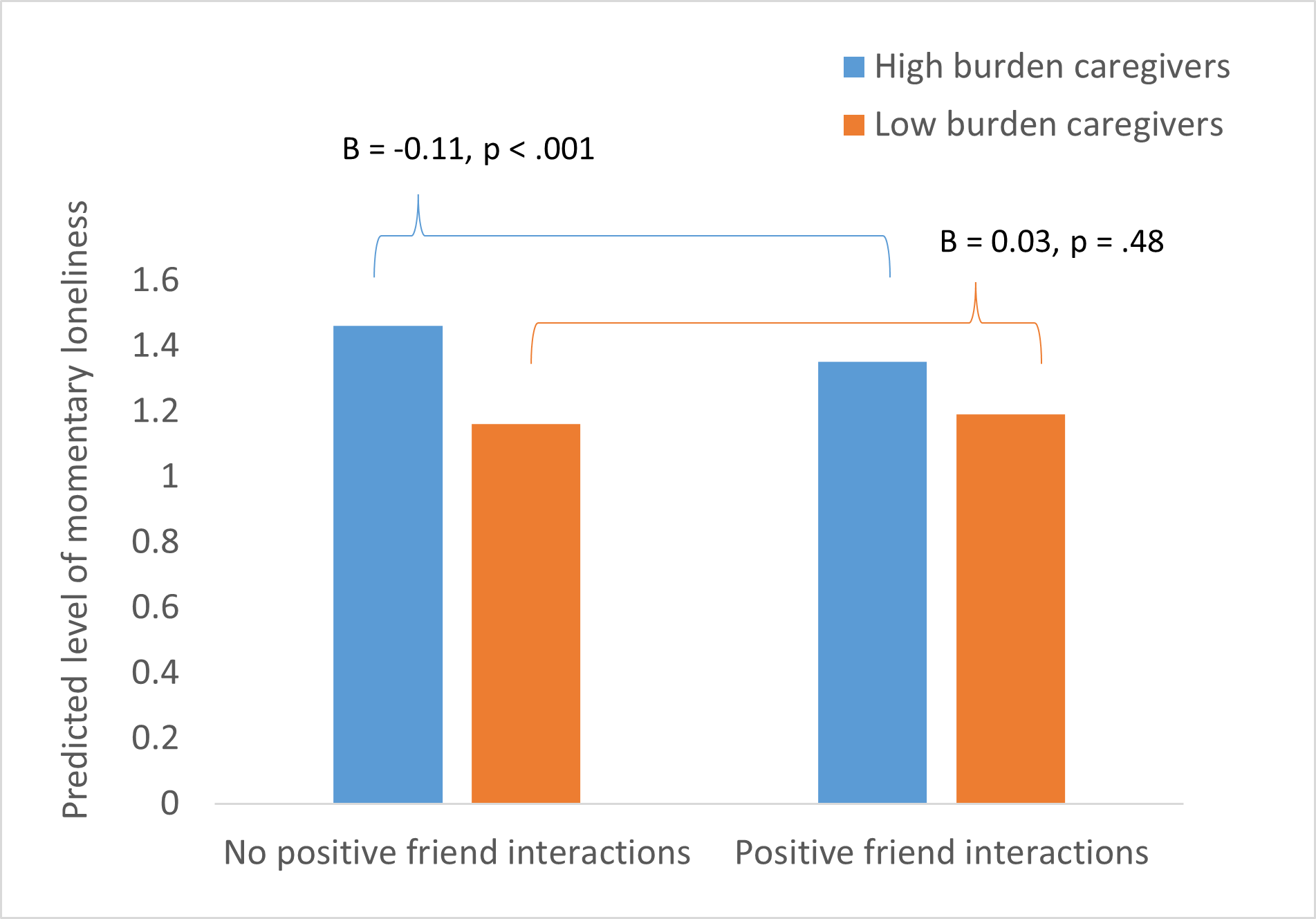


**Supplementary Figure 2.** *The within-person effect of interactions with non-close friends on momentary loneliness by caregiving burden*


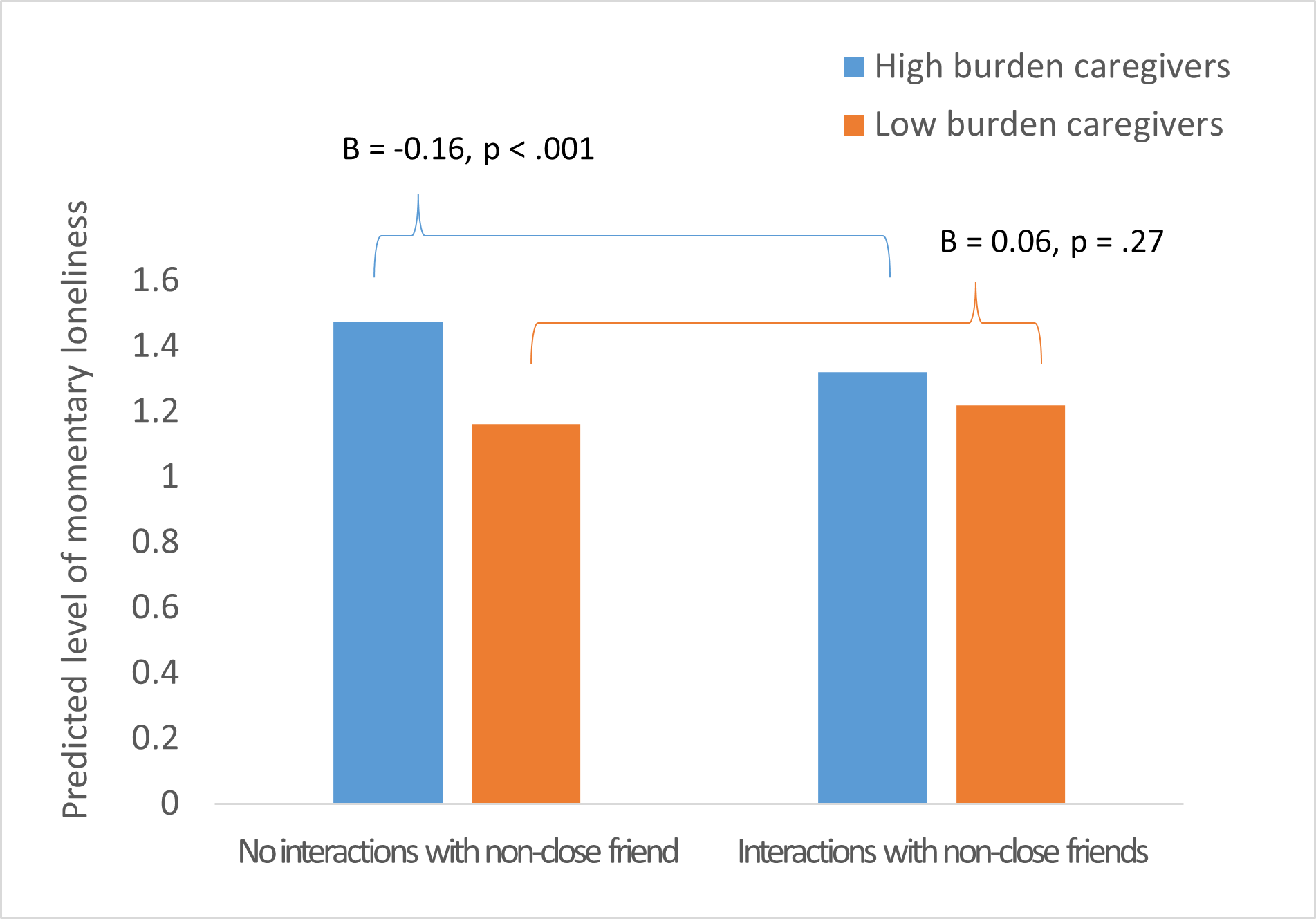

Supplement: gbaf190_Supplementary_Data [file gbaf190_supplementary_data.zip › JGSS suppl Ng, Turkelson, Kratz, & Birditt.docx]
